# Supplementary material for: METACASPASE8 (MC8) Is a Crucial Protein in the LSD1-Dependent Cell Death Pathway in Response to Ultraviolet Stress
Source: Int J Mol Sci. 2024 Mar 11;25(6):3195. doi: 10.3390/ijms25063195 (PMC10970217; doi:10.3390/ijms25063195)
Supplement: Supplementary file 1 [file ijms-25-03195-s001.zip › MC8 supplementary data Bernacki et all.pdf]

Supplementary figure and table

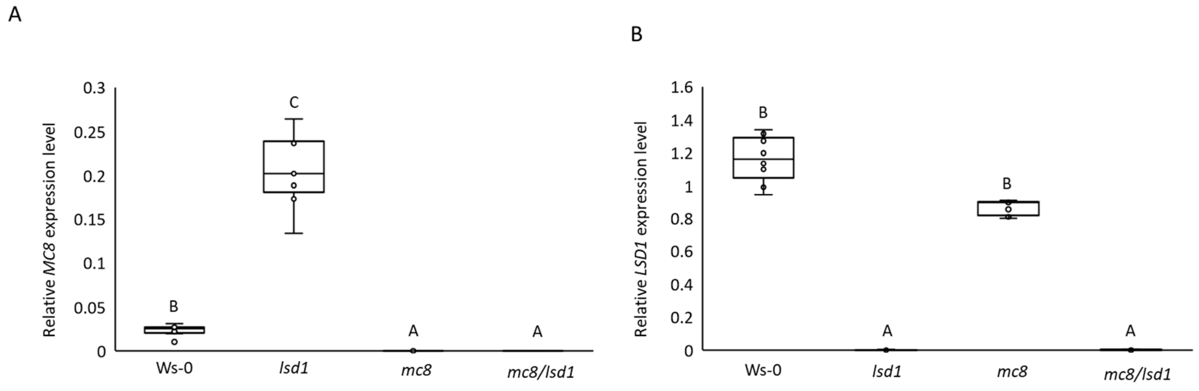

Figure supplementary S1. *MC8* (A) *and LSD1* (B)- relative expression in mutant tested during this study. Within a subgraph, values sharing common letters are not significantly different from each other ($p > 0.001$) ($n = 9$).

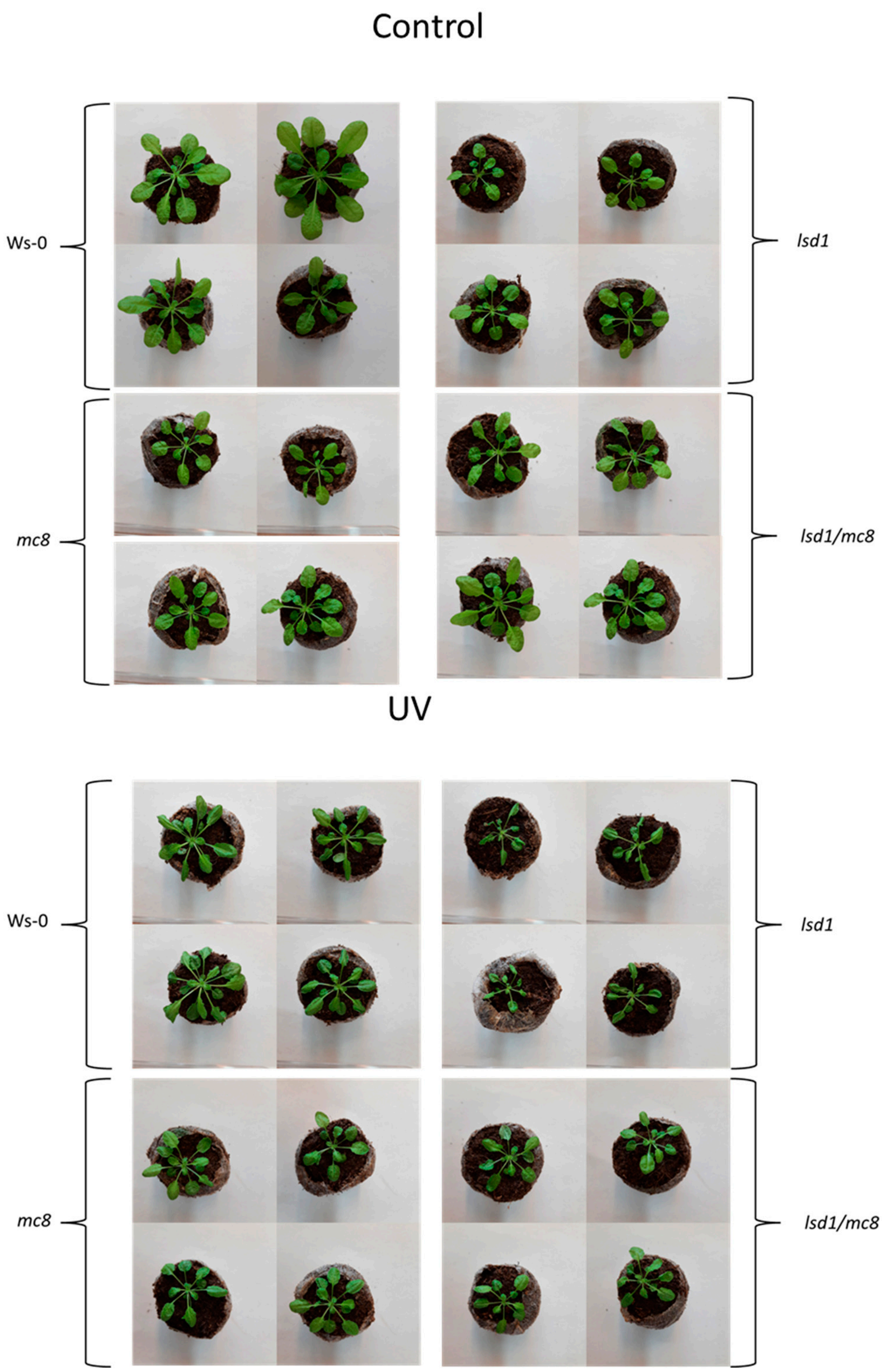

Figure supplementary S2. Plants morphology in tested mutants before (top) and after (bottom) UV irradiation.

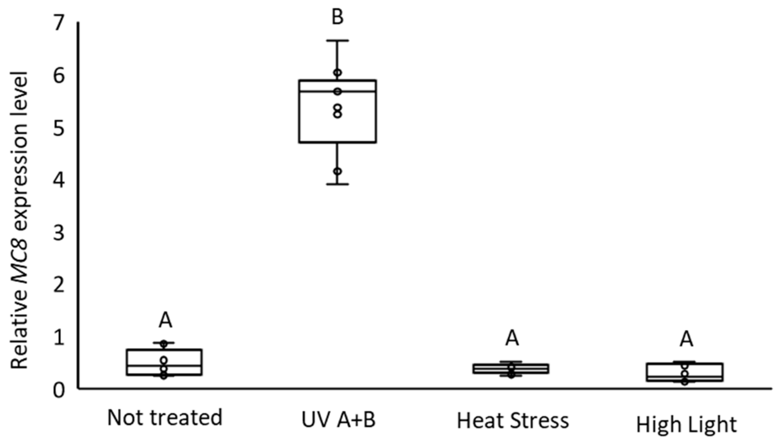

Figure supplementary S3. Expression of *AtMC8* under the influence of selected abiotic stresses. Within a subgraph, values sharing common labels (letters) are not significantly different from each other (p > 0.001) (n = 8–12).

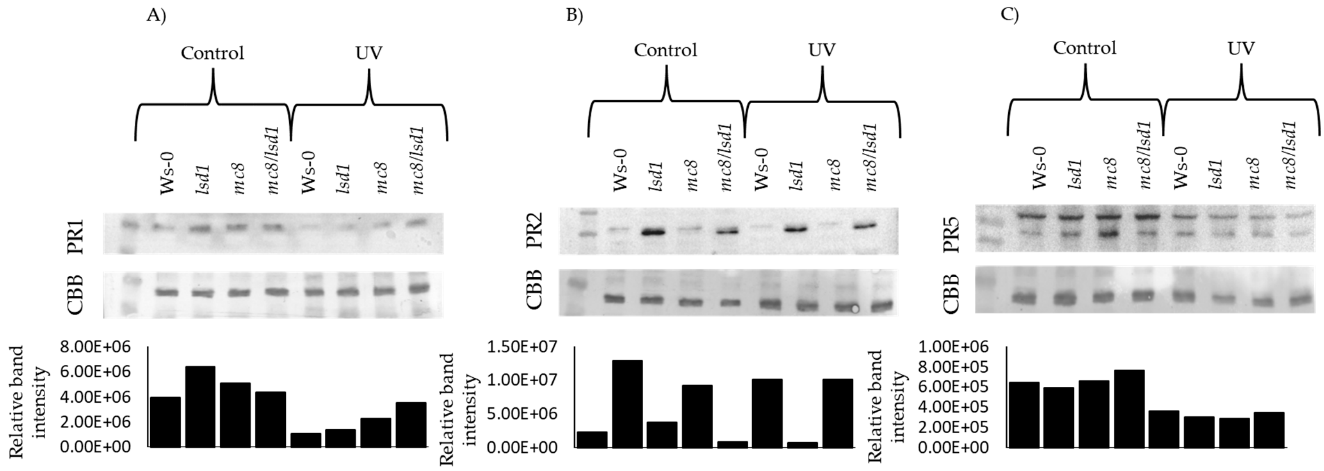

Figure supplementary S4. PR-proteins are degraded in response to UV irradiation. Western blot analysis of (A) PR1, (B) PR2 and (C) PR5 proteins in all tested genotypes in control condition and 24 h after UV irradiation. As loading controls CBB staining was used.

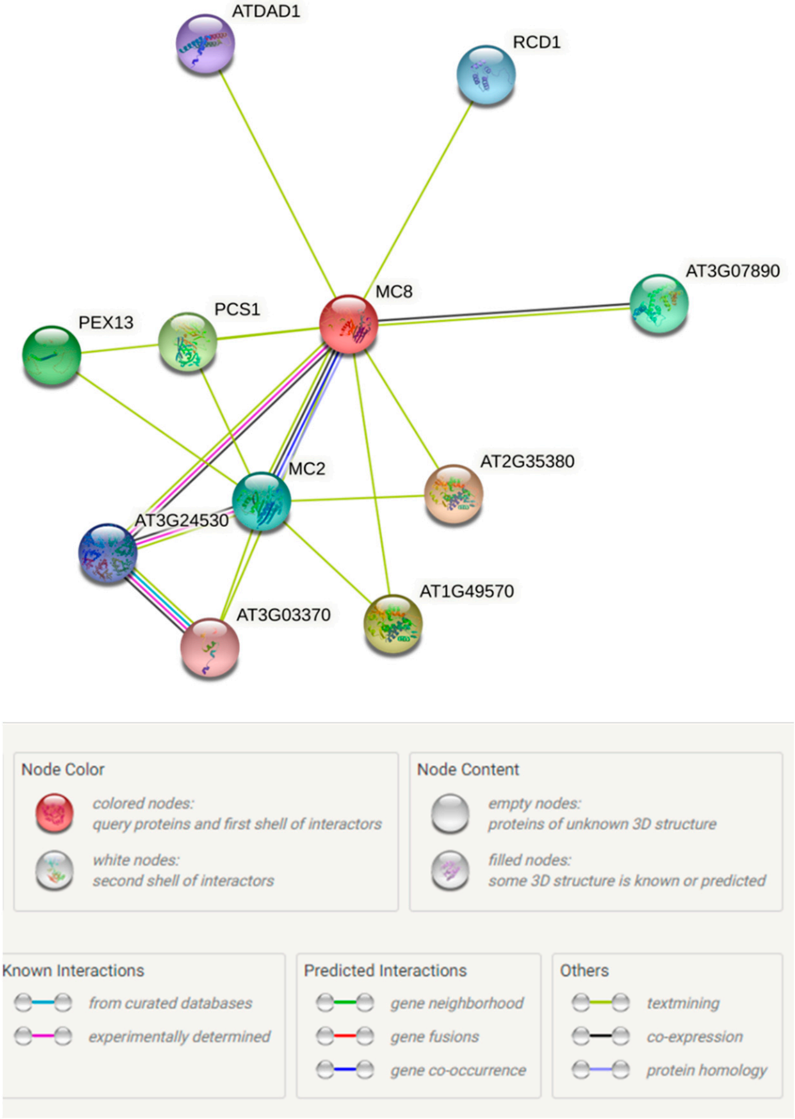

Figure supplementary S5. Predicted METACASPASE 8 interaction using STRING (string-db.org)

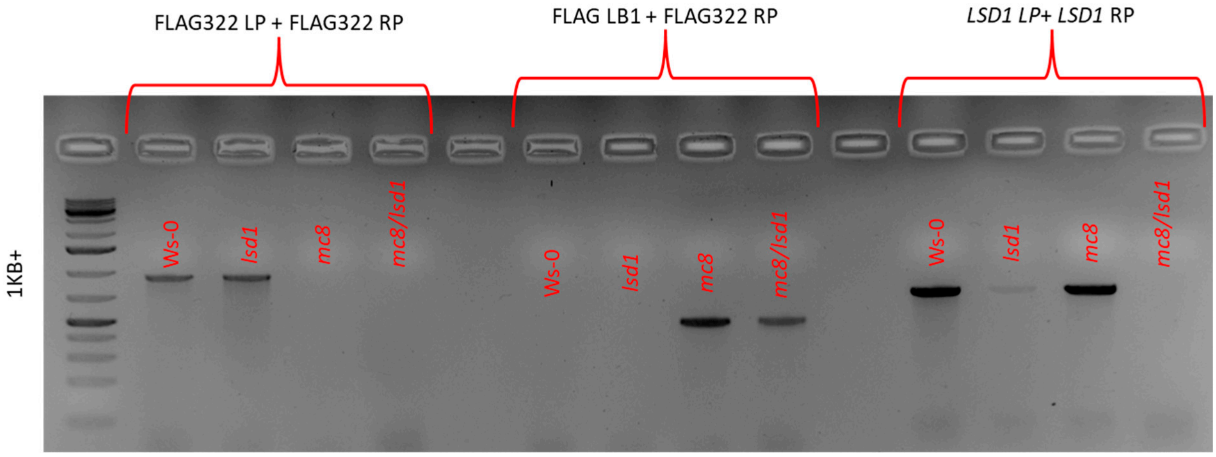

Figure supplementary S6. Genotyping of mutants used in this study.
